# The macrophage cytoskeleton acts as a contact sensor upon interaction with *Entamoeba histolytica* to trigger IL-1β secretion

**DOI:** 10.1371/journal.ppat.1006592

**Published:** 2017-08-24

**Authors:** Joëlle St-Pierre, France Moreau, Steve Cornick, Jeanie Quach, Sharmin Begum, Luz Aracely Fernandez, Hayley Gorman, Kris Chadee

**Affiliations:** Department of Microbiology, Immunology and Infectious Diseases, Snyder Institute for Chronic Diseases, University of Calgary, Calgary, Alberta, Canada; University of Virginia Health System, UNITED STATES

## Abstract

*Entamoeba histolytica* (*Eh*) is the causative agent of amebiasis, one of the major causes of dysentery-related morbidity worldwide. Recent studies have underlined the importance of the intercellular junction between *Eh* and host cells as a determinant in the pathogenesis of amebiasis. Despite the fact that direct contact and ligation between *Eh* surface Gal-lectin and *Eh*CP-A5 with macrophage α_5_β_1_ integrin are absolute requirements for NLRP3 inflammasome activation and IL-1β release, many other undefined molecular events and downstream signaling occur at the interface of *Eh* and macrophage. In this study, we investigated the molecular events at the intercellular junction that lead to recognition of *Eh* through modulation of the macrophage cytoskeleton. Upon *Eh* contact with macrophages key cytoskeletal-associated proteins were rapidly post-translationally modified only with live *Eh* but not with soluble *Eh* proteins or fragments. *Eh* ligation with macrophages rapidly activated caspase-6 dependent cleavage of the cytoskeletal proteins talin, Pyk2 and paxillin and caused robust release of the pro-inflammatory cytokine, IL-1β. Macrophage cytoskeletal cleavages were dependent on *Eh* cysteine proteinases *Eh*CP-A1 and *Eh*CP-A4 but not *Eh*CP-A5 based on pharmacological blockade of *Eh* enzyme inhibitors and *Eh*CP-A5 deficient parasites. These results unravel a model where the intercellular junction between macrophages and *Eh* form an area of highly interacting proteins that implicate the macrophage cytoskeleton as a sensor for *Eh* contact that leads downstream to subsequent inflammatory immune responses.

## Author summary

The protozoan parasite *Entamoeba histolytica* can establish an enteric infection in human hosts that leads to symptoms ranging from diarrhea to abscesses in the liver and the brain. Host susceptibility to amebic infection is in part determined by the quality and potency of the host immune response that occurs once the parasite overcomes the mucus bilayers and colonic epithelial barriers, and invades underlying tissues. At the cellular level, one of the key events that shape the inflammatory response occurs during direct parasite interaction with host macrophages via surface proteins. The ensuing cascades of intracellular signaling events have only partly been uncovered. Interestingly, only direct interaction between live parasites and macrophages, as opposed to soluble factors or dead parasites, is a prerequisite to the generation of a prompt raging pro-inflammatory response. We have sought to further elucidate the mechanisms by which macrophages distinguish live parasites and found that the macrophage cell skeleton undergoes rapid significant alteration upon *Eh* contact. Furthermore, we uncovered a previously unknown role for two *Eh* enzymes in triggering macrophage pro-inflammatory responses. Through this work, we gain a better understanding of the molecular interactions that occur at the macrophage-ameba interface that regulate host inflammatory responses.

## Introduction

Amebiasis is caused by the protozoan parasite *Entamoeba histolytica* (*Eh*) and is estimated to affect approximately 50 million people worldwide [1]. It is a major cause of mortality and morbidity, particularly in children of developing countries. *Eh* carriers remain asymptomatic in most cases, but in approximately 10% of infected individuals, *Eh* invades the intestinal tissues and triggers symptoms such as diarrhea, dysentery and colitis [2]. *Eh* can also migrate to other soft tissue organs, notably the liver where it causes abscesses, and can lead to death. The reasons that lead to the development of symptomatic infection are not fully defined but host genetic makeup, quality of the immune response and *Eh* expression of virulence factors are thought to be contributing factors. For example, polymorphisms in the leptin gene, as well as expression of certain HLA class II alleles, have been associated with increased vulnerability to amebiasis [3–6]. At the parasite level, expression of many virulence factors by *Eh* have been shown to modulate the host inflammatory response as well as the ability to invade soft tissue organs outside the gut [7, 8]. *Eh* binding to the mucus layers and host cells is mediated by its major surface component, the Gal/GalNAc lectin (Gal-lectin) [9, 10]. This 170kDa cell surface molecule has been shown to trigger innate immune responses and is a major target of the adaptive immune response [11–16]. Through homology search of the *Eh* genome, at least 50 genes encoding for cysteine peptidases (CP) have been identified [17, 18]. Of these, *Eh*CP-A1, *Eh*CP-A2 and *Eh*CP-A5 are the most highly expressed in axenically cultured *Eh* isolate HM-1:IMSS and account for the majority of cysteine proteinase activity *in vitro* [17, 19]. *Eh*CP-A5 is expressed on the cell surface, *Eh*CP-A2 localizes to the internal and external cell membrane and *Eh*CP-A1 localizes to intracellular vesicles [20–22]. These CPs have been shown to play a role in the pathogenesis of amebiasis [20, 23–25]. Moreover, as these CPs are required for *Eh* life cycle and are key virulence factors, they represent attractive pharmaceutical targets [26].

The immune response is also a major determinant of the capacity of *Eh* to cause disease [2, 7, 8]. Upon invasion of underlying gut tissue, *Eh* are met by gut resident macrophages. These cells have been shown to secrete high amounts of pro-inflammatory cytokines such as TNF-α and IFN-γ and possess amebicidal activity through the release of nitric oxide [11, 27]. TNF-α secretion has been associated with increased diarrheal disease, whereas nitric oxide and IFN-γ are protective [28]. We have shown that macrophages only secrete IL-1β upon direct contact with *Eh* [16, 29] that was dependent on initial adhesion by *Eh* Gal-lectin and engagement of macrophage α_5_β_1_ integrin by *Eh* cysteine protease 5 (*Eh*CP-A5) RGD sequences that activated the NLRP3 inflammasome. Thus, there is growing evidence that signaling events initiated at the intercellular junction between *Eh* and macrophages shape the ensuing immune response that are central in determining host susceptibility. We hypothesize that the macrophage cytoskeleton acts as a sensor that can distinguish the threat posed by invasion of live pathogens within the underlying gut tissue as opposed to soluble factors that bind to host immune cell receptors.

In this study, we sought to uncover the molecular mechanisms that allow macrophage recognition of invasive *Eh*. We found that live *Eh* in contact with macrophages induces caspase-6-dependent cleavage of cytoskeletal proteins triggered by *Eh*CP-A1 and *Eh*CP-A4 and culminates in IL-1β secretion by macrophages. These studies demonstrate a novel macrophage cellular pathway, as well as a novel function for parasite CPs in triggering IL-1β secretion upon contact with *Eh*. Moreover, this study further strengthens the hypothesis that direct contact between macrophages and *Eh* is a key event that initiates multiple cellular mechanisms at the intercellular junction that culminates in a potent pro-inflammatory response.

## Results

### Macrophage cytoskeleton undergoes rearrangement upon contact with *Eh*

**Fig 1:**
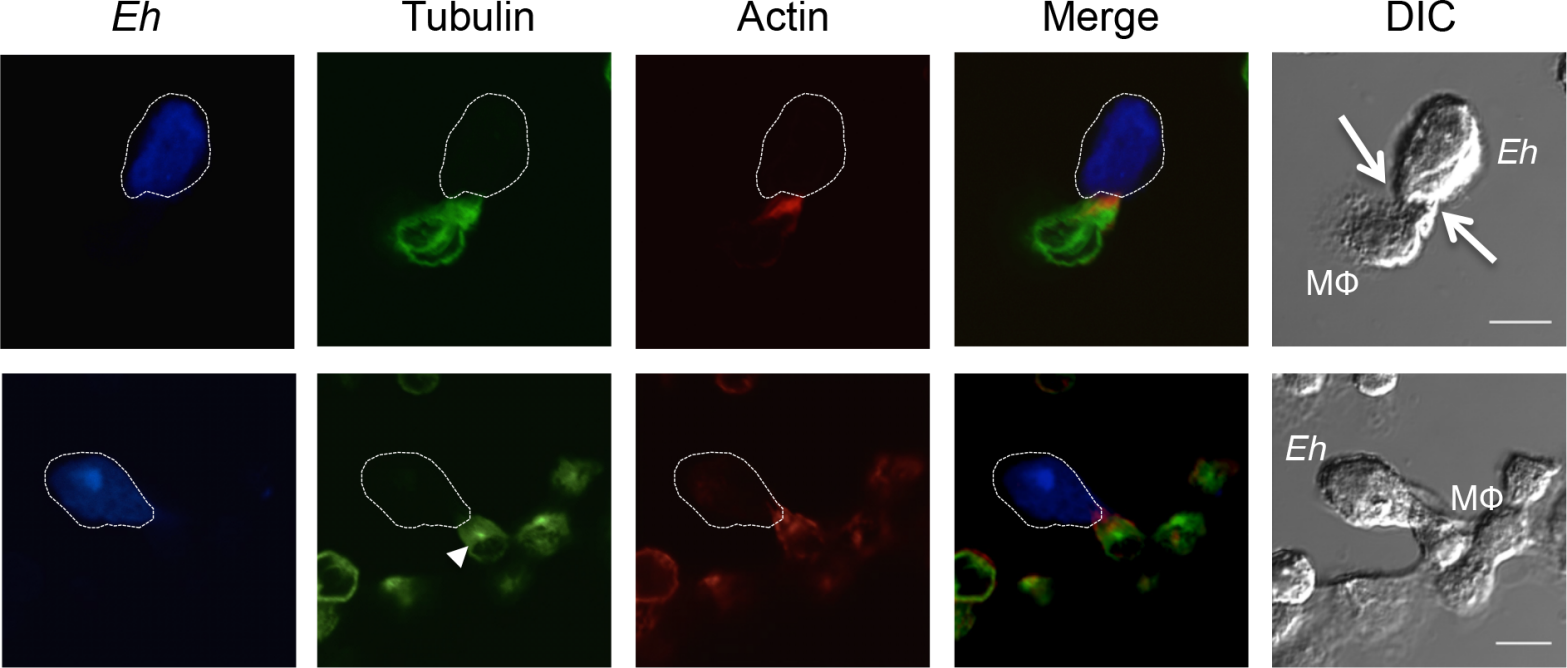
Cytoskeletal modulation in macrophages upon contact with *Eh*. Confocal microscopy of THP-1 macrophages incubated with *Eh* for 10 min. *Eh* were stained with Cell Tracker Blue prior to incubation. Cells were then fixed, permeabilized and stained with Alexa-488-conjugated anti-tubulin, or isotype control, and with Alexa-633-conjugated phalloidin. Images are representative of three independent experiments. The dotted white line indicates the position of *Eh* and the arrows in the DIC images shows the point of contact with the macrophage (MΦ). The arrowhead (tubulin frame in lower panel) shows the macrophage microtubule-organizing center. Scale bar 10μm.

Recent studies [16, 29, 30] have established that direct contact between host cells and *Eh* mediated through the Gal-lectin and engagement of host cell integrins by *Eh*CP-A5 at the intercellular junction is a critical initiation step in eliciting a robust pro-inflammatory response. These events require an intact actin cytoskeleton for recruitment of the NLRP3 inflammasome to contact sites as well as caspase-1 activation and IL-1β secretion [29]. *Eh* have been shown to distinguish between live and dead cells and adapt their capacity to interact with the host membrane, as underlined by the fact that the former triggers trogocytosis preferentially whereas the latter induces phagocytosis of host cells [31, 32]. These findings suggest that dynamic remodeling of the host cell cytoskeleton is probable upon direct contact with *Eh* and may have functional implications in terms of host cell, as well as parasite responses. To establish whether *Eh* induces cytoskeletal remodeling in macrophages upon direct contact, macrophages were incubated with *Eh* for 10 min, and the cytoskeletal proteins actin and tubulin were visualized by confocal microscopy. As predicted, both tubulin and actin were heavily concentrated at the macrophage-*Eh* contact site (**Fig 1**). Furthermore, microtubules were rapidly polarized towards the *Eh* contact site at the macrophage microtubule-organizing center. These results show that the cytoskeleton dynamically responded to *Eh* attachment at the macrophage intercellular junction.

### Cytoskeletal-associated proteins undergo cleavage upon contact with *Eh*

**Fig 2:**
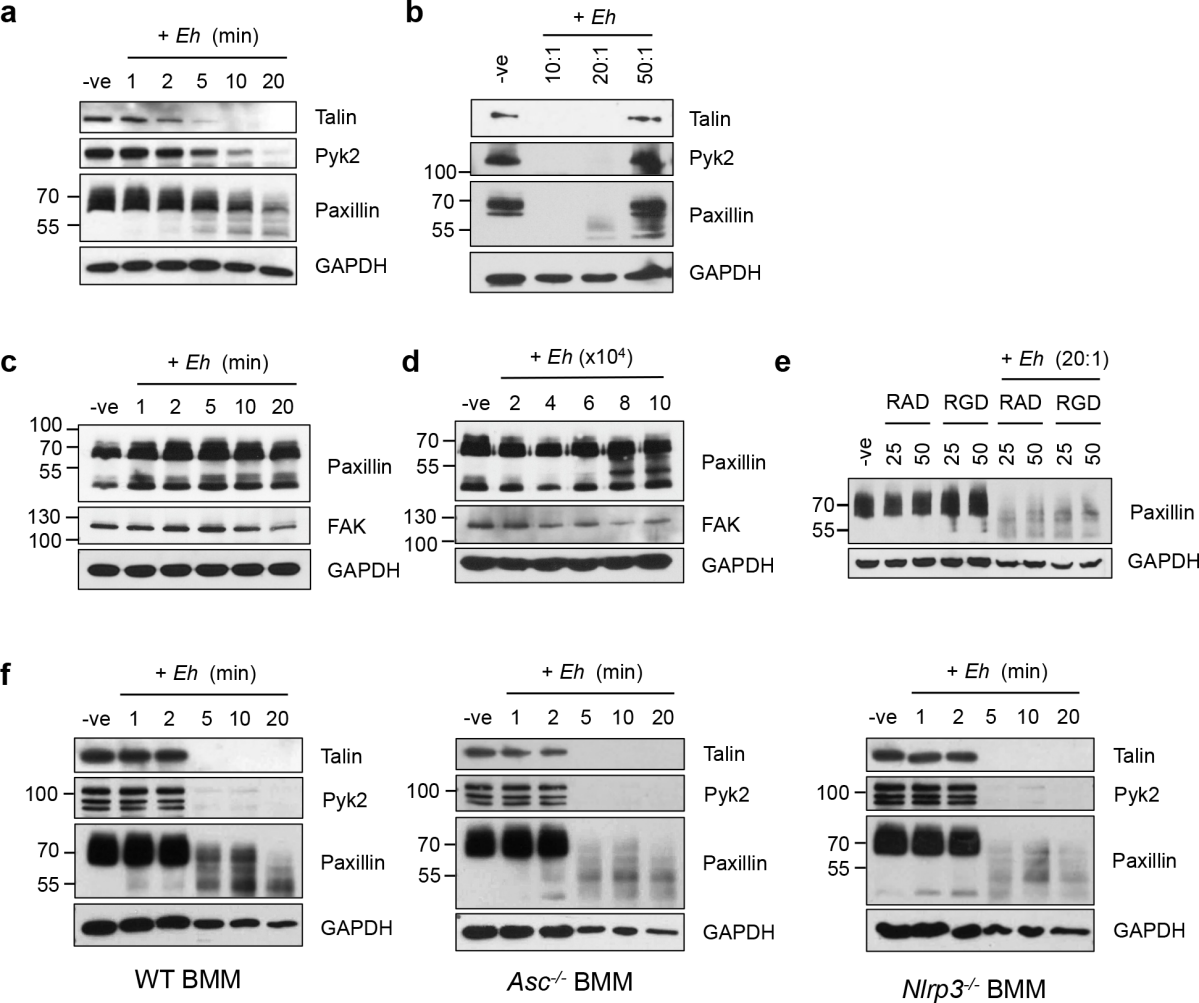
Degradation of cytoskeletal-associated proteins upon contact with *Eh* is observed in macrophages but not in colonic epithelial cells and is independent of inflammasome activation downstream of EhCP-A5 ligation to the α_5_β_1_ integrin. THP-1 macrophages were incubated for (**a**) increasing times with *Eh* (20:1 ratio), or (**b**) for 5 min with the indicated macrophage-to-*Eh* ratios. After incubation, cells were washed and lysed. Equal amounts of lysates were loaded onto SDS-PAGE gel and immunoblotted with the indicated antibodies. T84 colonic epithelial cells were grown to confluency in wells of 12-well plates and (**c**) incubated for increasing times with 4 x 10^5^ *Eh*, or (**d**) for 5 min with increasing amounts of *Eh*. (**e**) THP-1 macrophages were incubated for 5 min with the RGD small inhibitory peptide, or RAD as control, prior to stimulation with 20:1 *Eh* for 5 min, or left untreated (-ve). Bone marrow macrophages (BMM) derived from WT, *Asc*^*-/-*^ or *Nlrp3*^*-/-*^ mice were incubated with *Eh* (10:1) for the indicated amount of time prior to cell lysis (**f**). Lysates were loaded onto SDS-PAGE and immunoblotted with anti-Talin, anti-Pyk2, anti-paxillin and anti-GAPDH. Results are representative of more than three (**a, b, e, f**), or two independent experiments (**c, d**).

Cytoskeletal-associated proteins are intricately and extensively regulated by post-translational modification to regulate cytoskeletal dynamics during many cellular processes such as adhesion, cell death as well as cell-specific functions such as cytokine secretion. Previous studies have shown that *Eh* in contact with liver sinuisoidal endothelial cells triggers the breakdown of actin stress fibers, and relocalization of cytoskeletal-associated proteins [33, 34]. In the setting of macrophage-*Eh* interactions, phosphorylated paxillin and α_5_β_1_ integrin have been shown to concentrate at sites of contact between macrophages and *Eh* [29]. We investigated whether other macrophage cytoskeletal proteins were subjected to post-translational modifications and found that upon *Eh* contact with macrophages the degradation of at least three key cytoskeletal-associated proteins, talin, Pyk2 and paxillin occurred in a time- and dose-dependent manner (**Fig 2A and 2B**). Pyk2 is a signaling kinase mostly expressed in immune cells that triggers signaling downstream of integrins [35]. Paxillin and talin both act as scaffold proteins downstream of integrin signaling and play a key role in cytoskeletal remodeling [36, 37]. Appearance on western blot of a 53-kDa paxillin cleavage product occurred within 2 min suggesting that this is an early event during macrophage interaction with *Eh*. Although we originally postulated that paxillin would be phosphorylated upon contact with *Eh*, we were unable to assess the phosphorylation status of paxillin by western blot, as it was rapidly degraded. Interestingly, although *Eh* also engage integrins on the surface of T84 human colonic cells cytoskeletal cleavage was minimal, as appearance of paxillin cleavage products (~55 kDa) were only observed with high concentrations of *Eh* (**Fig 2C and 2D**). The 53-kDa and lower bands on the other hand, are similar to the main cleavage product observed in macrophages, but are only present with high concentrations of *Eh* (Fig 2D). As Pyk2 is not expressed at detectable levels in T84 colonic cells cleavage of its ortholog Focal Adhesion Kinase (FAK) was assessed and it was not degraded.

We have previously shown that *Eh* engages the α_5_β_1_ integrin at the surface of macrophages through *Eh*CP-A5 RGD sequences to promote NLRP3 inflammasome activation. Although many of the cytoskeletal proteins cleaved upon contact with *Eh* were found downstream of integrins, pre-treatment of macrophages with the RGD integrin α_5_β_1_ inhibitory peptide did not inhibit cleavage of paxillin using high concentrations of *Eh* (**Fig 2E** compared to **Fig 2B**). Furthermore, cleavage of macrophage cytoskeletal proteins was similar in WT BMM as well as in *Asc*^*-/-*^ BMM that lack the Nlrp3 adapter ASC, and in *Nlrp3*^*-/-*^ BMMs (**Fig 2F**). These results show that macrophage interaction with *Eh* triggers rapid and potent cleavage of cytoskeletal-associated proteins that is independent of the previously characterized *Eh*CP-A5/α_5_β_1_ integrin interaction [29].

### Degradation of cytoskeletal-associated proteins upon *Eh* contact is mediated by caspase-6

**Fig 3:**
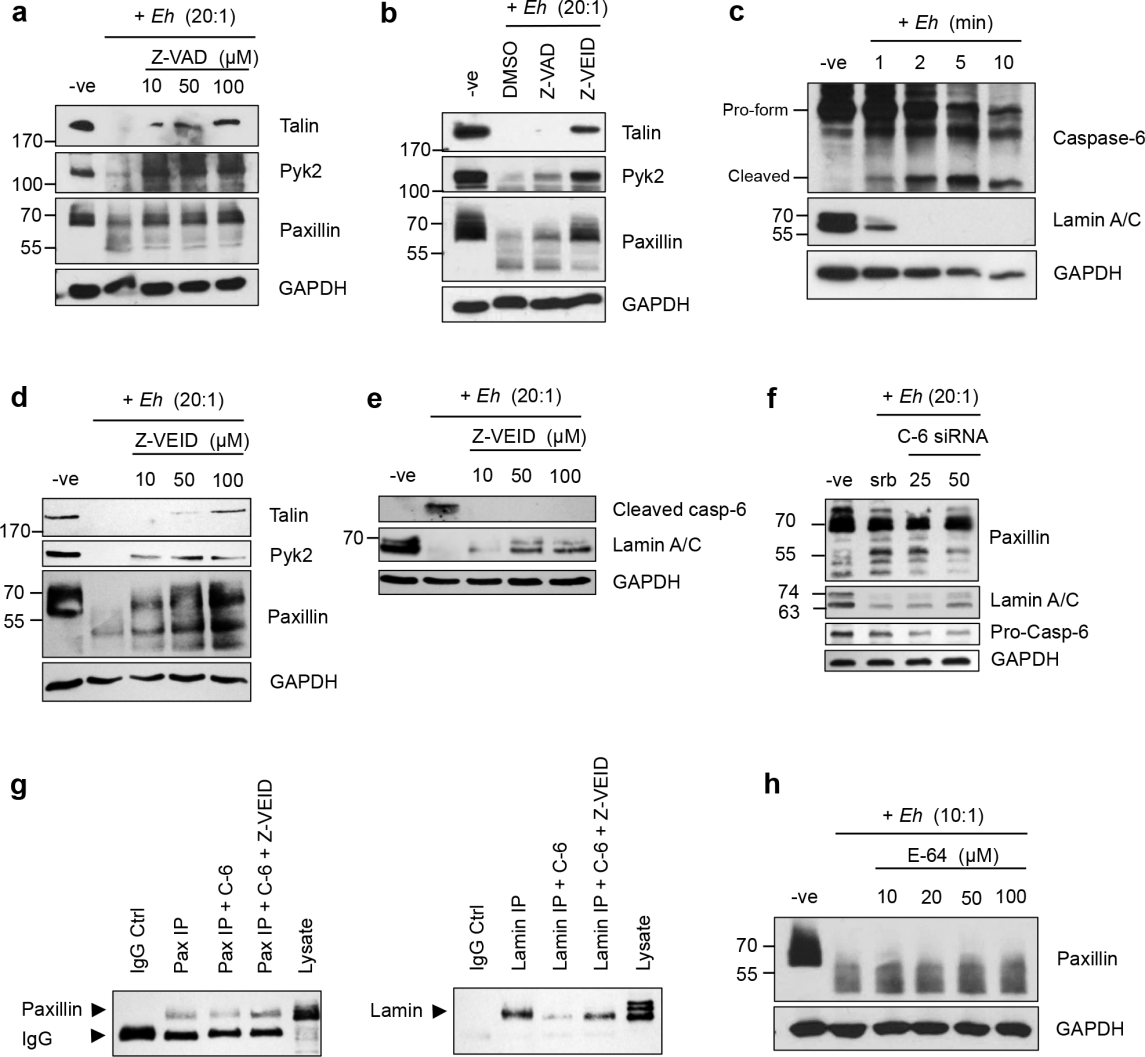
Degradation of macrophage cytoskeletal-associated proteins upon contact with *Eh* is mediated by caspase-6. (**a**) THP-1 macrophages were pre-treated with increasing amounts of the pan-caspase inhibitor Z-VAD-fmk or DMSO as control, for 1 h prior to incubation with *Eh* (20:1 ratio) for 5 min. Cleavage of the indicated cytoskeletal proteins was assessed by Western blot. (**b**) THP-1 macrophages were pre-treated with 50μM Z-VAD-fmk, 50μM of the caspase-6 inhibitor Z-VEID-fmk or DMSO as a control, followed by incubation with *Eh* (20:1) for 5 min. (**c**) Immunoblot of caspase-6 and its lamin A/C in macrophages incubated with *Eh* (20:1) for the increasing incubation times. Dose-dependent inhibition of cleavage of the indicated cytoskeletal proteins (**d**) or caspase-6 and its substrate (**e**) by Z-VEID. To inhibit calpain activation in macrophages, cells were pretreated with E-64 prior to incubation with *Eh* (10:1) and cell lysis (**f**). Lysates were loaded onto SDS-PAGE and immunoblotted with the indicated antibodies. (**g**) THP-1 cells were transfected with 25 and 50nM of caspase-6 siRNA, or scramble siRNA. After 48 h, transfected cells were stimulated for 2 min with *Eh*, followed by Western blot with the indicated antibodies. (**h**) Direct cleavage of paxillin was assessed by incubated immunoprecipitated paxillin with recombinant caspase-6 (C-6), with or without Z-VEID. Immunoprecipitated lamin was used as a positive control.

Paxillin has been shown to be susceptible to proteolytic cleavage by calpains and caspase-3 in the context of adhesion regulation and apoptosis [38–40]. Similarly, calpain-mediated cleavage of talin and Pyk2 is necessary for formation and disassembly of focal adhesions in osteoclasts [41]. Furthermore, both talin and paxillin have been shown to be cleaved by calpains in Jurkat T-cells incubated with *Eh* [42]. Accordingly, we next investigated whether cleavage of these cytoskeletal proteins were mediated by proteolytic cleavage by calpains or caspases. To do this, macrophages were pre-treatment with the pan-caspase inhibitor Z-VAD-fmk and in response to *Eh*, inhibited the cleavage of talin, Pyk2 and paxillin in a dose-dependent manner (**Fig 3A**). Through the use of a caspase inhibitor panel, we next identified the specific caspase responsible for cleavage of these proteins as caspase-6 (**Fig 3B**). None of the other inhibitors specific for caspases-1, -2, -3, -5, -8, and -9 inhibited *Eh-*induced cleavage of cytoskeletal-associated proteins. Consistent with these findings we found that caspase-6 was rapidly activated in *Eh* contacted macrophages, as shown by the appearance of the cleaved form of caspase-6 and degradation of one of its substrates, lamin (**Fig 3C**). Predictably, cleavage of macrophage cytoskeletal proteins was inhibited in a dose-dependent manner by the caspase-6-specific inhibitor Z-VEID (**Fig 3D**). Similarly, cleavage and activation of caspase-6 and its substrate lamin were also inhibited with increasing concentrations of Z-VEID upon macrophage stimulation with *Eh* (**Fig 3E**). To confirm specificity for caspase-6 involvement in paxillin degradation, we silenced caspase-6 by siRNA in THP-1 cells and used scramble siRNA as a control, followed by incubation with *Eh* for 2 min. As predicted, silencing caspase-6 inhibited the degradation of paxillin cleavage products (proteins >55 kDa) with increasing concentration of caspase-6 siRNA (**Fig 3F**). Cleavage of lamin was used as a positive control that was also inhibited from degradation with increasing concentration of caspase-6 siRNA as compared to the negative and scrambled siRNA controls. This data, together with the inhibition of paxillin by treatment with Z-VEID, suggests that *Eh*-induced cytoskeletal cleavage in macrophages was dependent on caspase-6 activation. To determine whether paxillin is a direct substrate of caspase-6, we assessed whether caspase-6 was able to cleave paxillin *in vitro* by incubating paxillin immunoprecipitated from THP-1 cells with recombinant human caspase-6. We found that recombinant caspase-6 was able to cleave immunoprecipitated paxillin, and that this cleavage was inhibited by treatment with Z-VEID (**Fig 3G**). The bottom band corresponds to mouse IgG from the immunoprecipitate, which cross-reacts with the secondary antibody used for western blot and served as a negative control for caspase-6 cleavage. A similar pattern was observed with the known caspase-6 substrate lamin (**Fig 3G**). To assess whether calpains also participated in the cleavage of the cytoskeletal protein paxillin, macrophages were pre-treated with increasing concentrations of E-64 that inhibits cysteine proteases as well as calpains, and it did not inhibit cleavage upon incubation with *Eh* (**Fig 3H**).

### Cytoskeletal remodeling is dependent on contact with live *Eh*

**Fig 4:**
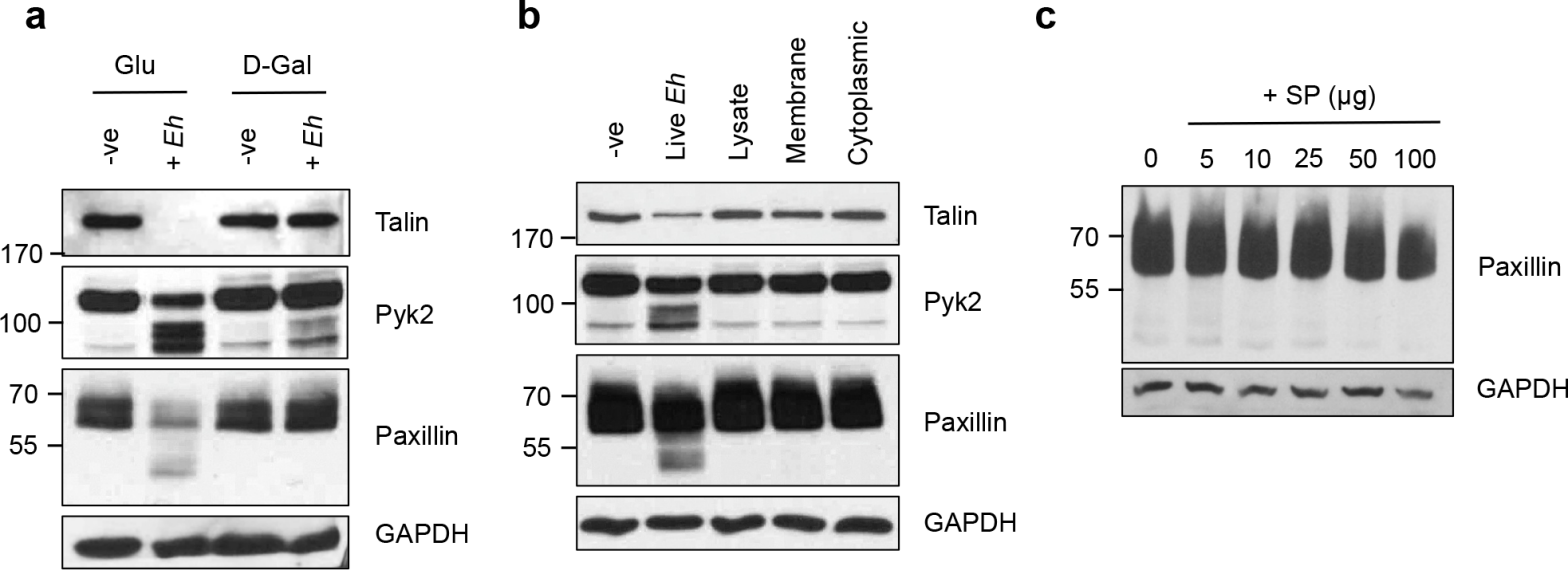
Macrophage cytoskeleton remodeling is dependent on direct contact with live *Eh*. (**a**) *Eh* were pre-treated for 5 min with 55mM D-galactose, or glucose as an osmotic control prior to incubation with macrophages for 5 min at a 20:1 ratio. After incubation, cells were washed, lysed and equal amounts of lysates were loaded onto SDS-PAGE gel and immunoblotted with the indicated antibodies. (**b**) THP-1 cells were stimulated for 5 min with live *Eh* at a 20:1 ratio, whole lysates of *Eh*, or with membrane or cytoplasmic fractions of an equal amount of *Eh*. Lysates were analyzed as in (**a**). (**c**) Increasing amounts of *Eh* secreted/excreted proteins (SP) were incubated with macrophages for 15 min. Equal amounts of lysates were loaded onto SDS-PAGE and analyzed by Western blot with anti-paxillin and anti-GAPDH.

We next investigated the *Eh* requirements for triggering cleavage of the cytoskeletal-associated proteins. Gal-lectin is the major surface adhesin expressed by *Eh* and is necessary for binding macrophages [10, 43]. Predictably, inhibited *Eh* binding to macrophages with galactose completely abrogated cleavage of the cytoskeletal proteins talin, Pyk2 and paxillin whereas glucose as an osmotic control had no inhibitory effect (**Fig 4A**). We next evaluated whether stimulation of macrophages with subcellular fractions of *Eh* was capable of triggering cleavage of the cytoskeletal-associated proteins, as to further assess and identify the *Eh* virulence factors responsible for this mechanism. Cleavage of talin, Pyk2 and paxillin was only observed upon stimulation of macrophages with live *Eh* but not with fresh lysates of whole *Eh*, the membrane fraction or the cytoplasmic fraction derived from equivalent numbers of *Eh* (**Fig 4B**), or with increasing concentrations of *Eh* excreted/secreted soluble proteins (SP) (**Fig 4C**). These results indicate that contact with live *Eh* to macrophages was critical and necessary to initiate cytoskeletal cleavage. This could be due to the necessity of the dynamic engagement of multiple receptors during contact between *Eh* and macrophages, as supported by our observations for *Eh*CP-A5 and Gal-lectin [16, 29]. Similarly, the process of trogocytosis by *Eh* has been shown to preferentially occur upon contact with live host cells [31, 32].

### *Eh*CP-A1 and *Eh*CP-A4 mediate cleavage of cytoskeletal-associated proteins

**Fig 5:**
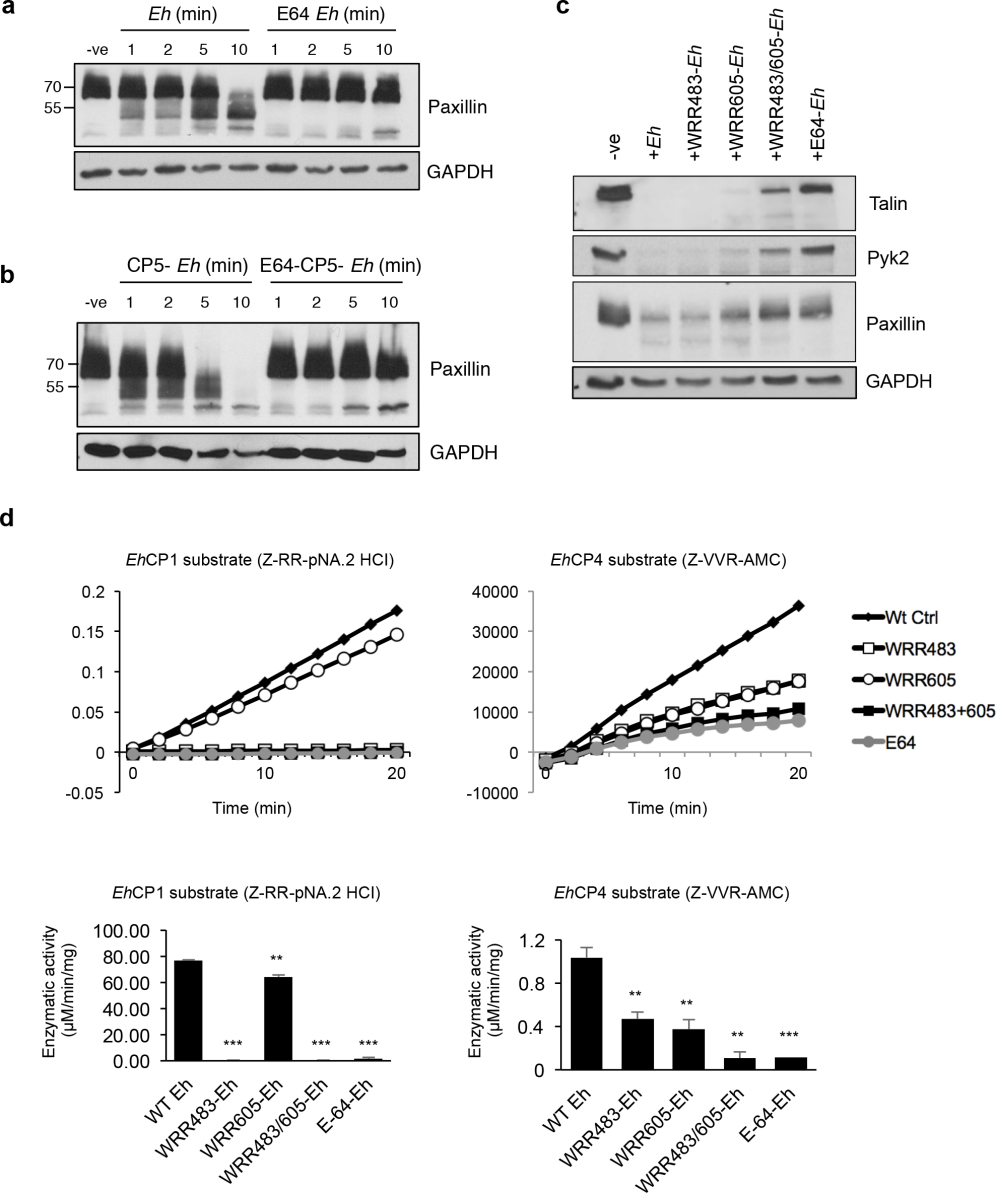
Inhibition of both *Eh*CP-A1 and *Eh*CP-A4, but not *Eh*CP-A5 prevents cleavage of macrophage cytoskeletal proteins. (**a**) Macrophages were incubated with wild-type *Eh* or E64-treated *Eh* (20:1) for the indicated amount of time. Cleavage of paxillin was assessed by Western blot. (**b**) Macrophages were incubated with CP-A5-deficient *Eh* or E64-treated CP-A5-deficient *Eh* (20:1) for the indicated amount of time. (**c**) Macrophages were stimulated with *Eh* pre-incubated 30 min with an inhibitor to *Eh*CP-A1 (WRR483), *Eh*CP-A4 (WRR605), both, or with E-64 at a 20:1 ratio for 5 min. (d) Enzymatic specificity of WRR inhibitors was evaluated by incubating inhibitor-treated *Eh* with *Eh*CP-A1 or *Eh*CP-A4 substrates for 20 min. Enzymatic activity for each substrate in the presence of inhibitors was calculated in μM/min/mg and shown in the bottom panel. Data are from three independent experiments and statistical significance was calculated with Student’s t-test (***p<0.0001, **p<0.005).

To assess whether *Eh*CP activity was necessary for cleavage of macrophage proteins, *Eh* were pre-treated overnight with the CP inhibitor E-64. Not surprisingly, E-64 treated *Eh* completely inhibited cleavage of cytoskeletal-associated proteins, indicating a role for *Eh* cysteine proteinase(s) in triggering caspase-6 activation and cleavage of paxillin (**Fig 5A**). To determine whether *Eh*CP-A5, a cell-surface CP that has been shown to directly bind integrins on the surface of epithelial cells and macrophages [29, 30], plys a role in this process we exposed macrophages to *Eh*CP-A5 deficient *Eh* and they were efficient at triggering the cleavage of paxillin that was inhibited in E64 treated *Eh*CP-A5 parasites (**Fig 5B**). This mechanism thus appears to be independent of the previously identified interaction between *Eh*CP-A5 and α_5_β_1_.

Based on these results, we next set out to identify the *Eh* cysteine proteases responsible for activating caspase-6 and subsequent cleavage of macrophage cytoskeletal proteins. Inhibitors to *Eh*CP-A1 and *Eh*CP-A4 (WRR483 and WRR605, respectively) have been carefully designed and synthetized to specifically inhibit their activity both *in vitro* and *in vivo* [20, 44]. *Eh*CP-A1, along with *Eh*CP-A5, is unique as they are not found in non-invasive *E*. *dispar* [19]. *Eh*CP-A1 has been shown to be highly transcribed *in vitro* and released by cultured trophozoites [17, 23, 45]. Inhibition of *Eh*CP-A1 with the WRR483 inhibitor has been shown to reduce *Eh* invasion in human intestinal xenografts in SCID mice [20]. *Eh*CP-A4 transcription levels, on the other hand, are low *in vitro*, however it has been shown to be one of the most up-regulated CPs during *Eh* cecal infection in mice [17, 46]. Fittingly, administration of WRR605 to *Eh*-infected mice has been shown to significantly reduce *Eh* burden and decrease inflammation [44], however the underlying molecular mechanisms remain unknown. To determine whether *Eh*CP-A1, *Eh*CP-A4, or both, were involved in mediating cleavage of macrophage cytoskeletal-associated proteins, we pre-treated *Eh* with either one of the synthetic inhibitors WRR483 and WRR605, or both, prior to incubation with macrophages. Pre-treatment of *Eh* with these inhibitors individually partially inhibited cleavage of talin, Pyk2 and paxillin in macrophages (**Fig 5C**). Whereas WRR483 did not inhibit the cleavage of any cytoskeletal protein, WRR605 partially rescued the cleavage of talin and Pyk2 and modestly paxillin. Interestingly, pre-treatment with both inhibitors had an additive effect and was similar to E64 treated *Eh* indicating that both *Eh*CP-A1 and *Eh*CP-A4 participated in cytoskeletal cleavage.

To confirm the specificity of the WRR483 and WRR483 inhibitors, we treated *Eh* trophozoites with either WRR483, WRR605, both, or E-64 as a control with known substrates to *Eh*CP-A1 (Z-RR) and *Eh*CP-A4 (Z-VVR). Degradation of Z-RR occurred in a linear fashion over time (**Fig 5D**, top panel). Enzymatic activity in the presence of the inhibitors is displayed in the bottom panel (**Fig 5D**). Differences in the units in the velocity and specific activity graphs are due to differences in the fluorogenic nature of the substrates used. As expected, the WRR483 inhibitor completely inhibited the degradation of the Z-RR substrate, and to a level similar to E-64, whereas WRR605 has a minimal effect (**Fig 5D**). Degradation of the known *Eh*CP-A4 substrate (Z-VVR), on the other hand, was inhibited by both WRR483 and WRR605 (**Fig 5D**). Together, they had an additive effect that was similar to the E-64 control. Whether this represents inhibition of *Eh*CP-A4 by WRR483, or whether *Eh*CP-A1 has specificity for the Z-VVR substrate remains to be determined, although the additive effect of both inhibitors suggest the later. Nevertheless, given that complete inhibition of paxillin cleavage is not seen with either inhibitor alone, but rather in an additive fashion when both inhibitors are present, suggest that both cysteine proteases are involved in triggering macrophage cytoskeletal-associated protein cleavage. The relative contribution of each cysteine protease remains to be further investigated with the use of recombinant proteins.

### *Eh*CP-A1 and *Eh*CP-A4 activate caspase-6 at the *Eh*-macrophage intercellular junction

**Fig 6:**
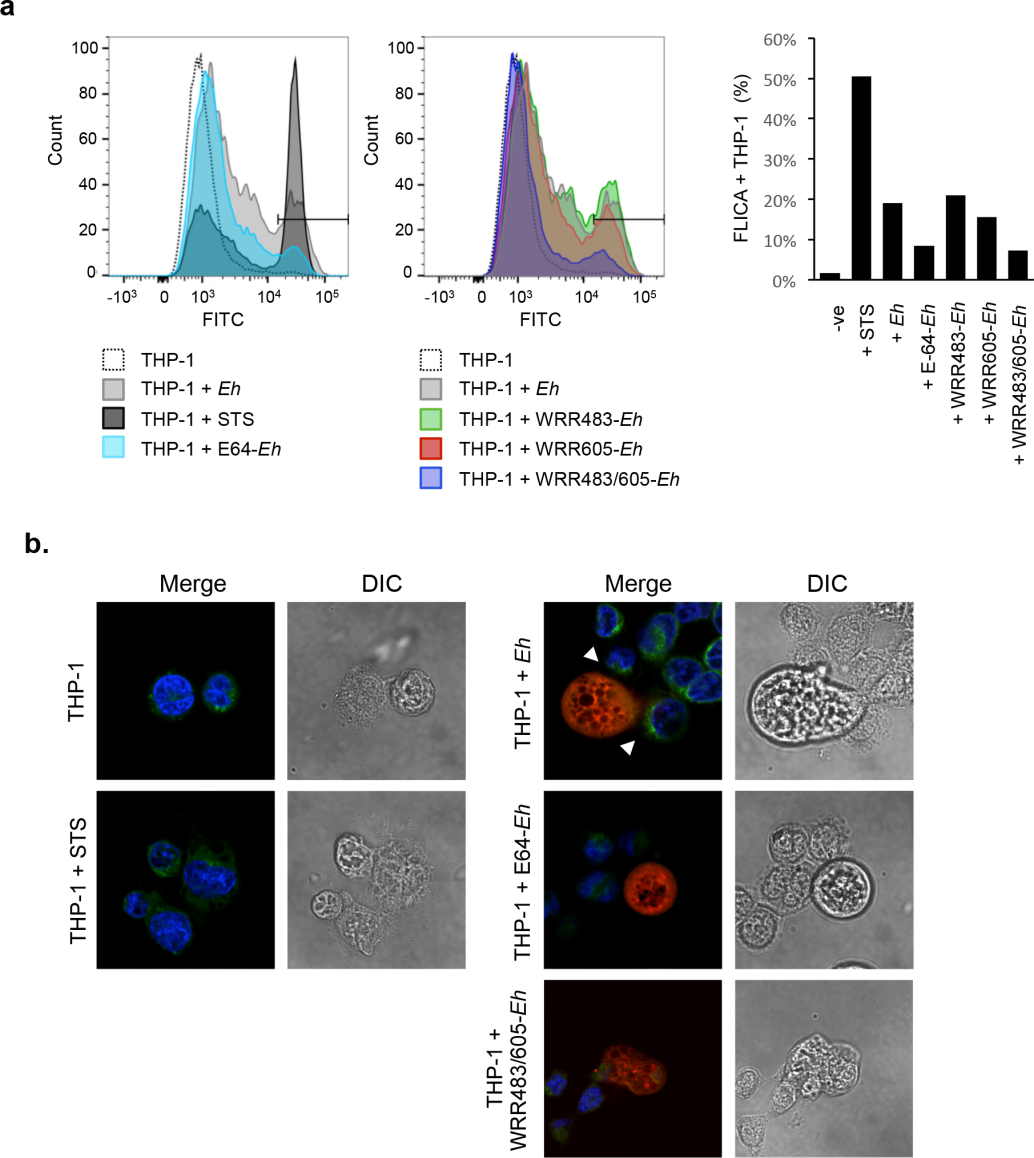
*Eh*-triggered caspase-6 activation is dependent on *Eh*CP-A1 and *Eh*CP-A4 and is localized at sites of contact. (**a**) Caspase-6 activation was measured by flow cytometry with the FITC-labeled caspase-6-FLICA probe in THP-1 macrophages incubated with 10:1 *Eh*, E64-treated *Eh*, CP-A5-deficient *Eh*, WRR483 pre-treated *Eh*, WRR605 pre-treated *Eh*, *Eh* pre-treated with both WRR483 and WRR605, left untreated, or with STS as a positive control. *Eh* were excluded from analysis based on forward and side scatter. Gating of percent positive cells was established according to the STS-positive population and percent positive cells for each treatment are indicated in the accompanying graph. (**b**) Caspase-6 activation for each treatment was also visualized by confocal microscopy using caspase-6-FLICA (green), DAPI (blue). *Eh* were pre-treated with Cell Tracker Orange (red) prior to incubation with THP-1 macrophages. STS was used as a positive control. Arrowheads show areas of polarization of caspase-6 towards the *Eh*-macrophage interface. The dotted white line indicates the position of *Eh* and the point of contact with the macrophage (MΦ). Results are representative of three independent experiments.

To quantify the involvement of *Eh*CP-A1 and *Eh*CP-A4 upstream of caspase-6 activation, we assessed caspase-6 activation by flow cytometry using a caspase-6-FLICA probe, which detects active caspase-6. To do this THP-1 macrophages were either left untreated or exposed to *Eh*, or treated with various *Eh*CP inhibitors (E-64, WRR483, WRR605, or both WRR483 and WRR605), or with staurosporine (STS) as a positive control for caspase-6 activation. Inhibition with E64 or with *Eh*CP-A1 and *Eh*CP-A4 in combination had the greatest effect on preventing caspase-6 activation (**Fig 6A**). We also assessed localization of caspase-6 with the FLICA probe by confocal microscopy. As envisaged, activated caspase-6 was strongly localized to regions within macrophages that were polarized towards *Eh* (**Fig 6B**). This polarization was disrupted in interactions with WRR483/605-treated *Eh*. STS was included as a positive control that shows diffuse caspase-6 activation within the macrophage cytoplasm (**Fig 6B**).

### *Eh*CP-A1 and *Eh*CP-A4 polarize to the macrophage-*Eh* interface

**Fig 7:**
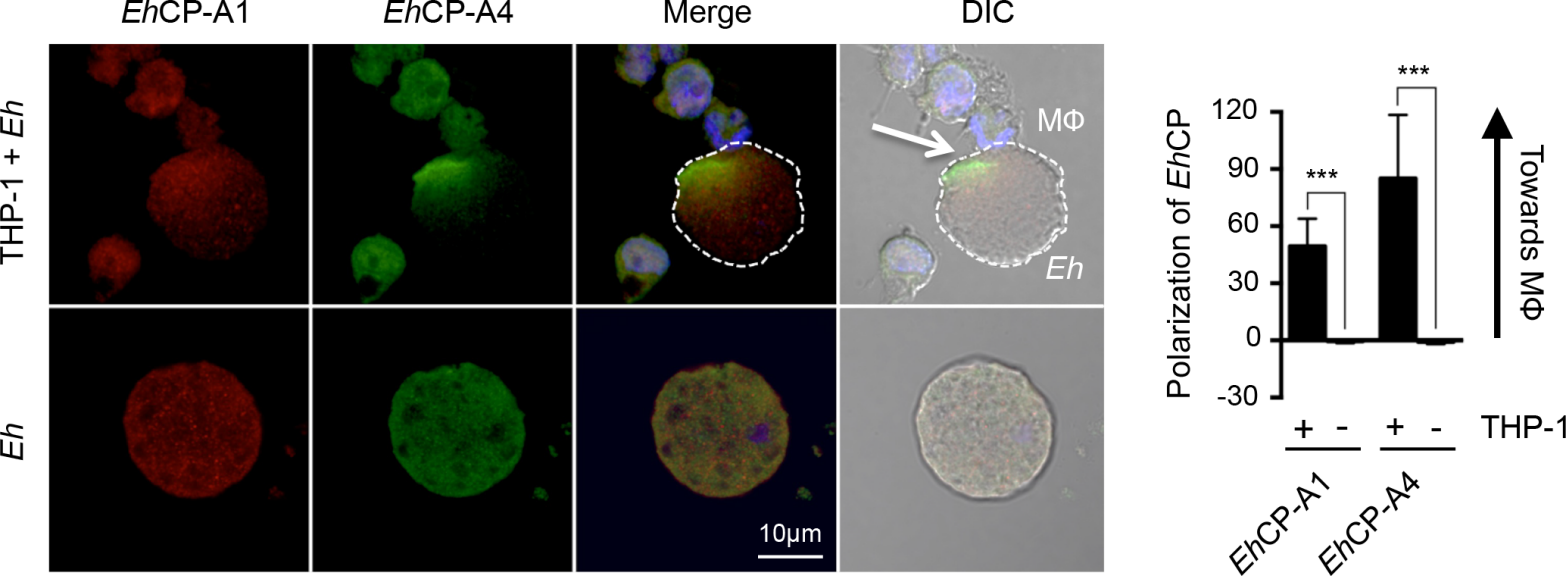
Cell surface *Eh*CP-A1 and *Eh*CP-A4 polarize to the macrophage contact site. To assess *Eh*CP-A1 and *Eh*CP-A4 localization, *Eh* were incubated with DAPI-treated THP-1 cells (top panels) or not (bottom panel). Slides were then stained with *Eh*CP-A1 and *Eh*CP-A4, and the appropriate secondary antibodies. *Eh*CP-A1 is shown in red, *Eh*CP-A4 is shown in green. Both *Eh*CP1 and *Eh*CP4 polarized on the *Eh* surface towards the macrophage whereas no polarization was observed in *Eh* basally (bottom panel). The dotted white line indicates the position of *Eh* and the arrow in the DIC images shows the point of contact with the macrophage (MΦ). Polarization of *Eh*CPs was quantified by comparing density of *Eh*CP staining at the leading half facing the macrophage in comparison to the tailing half. ***p<0.001 as determined by Student’s t-test.

Previous studies have shown that *Eh*CP-A1 is contained in cytoplasmic vesicles of trophozoites, whereas *Eh*CP-A4 localizes to intracellular vesicles, the nucleus and perinuclear endosplasmic reticulum [20, 44]. *Eh*CP-A1 is released upon stimulation of trophozoites with mucin whereas *Eh*CP-A4 is released upon contact of target cells [44, 47]. We sought to determine whether these cysteine proteinases are expressed on the *Eh* cell surface and whether they polarized to the macrophage-*Eh* interface. Cell-surface localization of these two amebic cysteine proteinases upon contact with macrophages was assessed by staining non-permeabilized cells. We found that in the absence of macrophages, *Eh*CP-A1 is diffusely distributed in a punctate-like pattern on the surface of *Eh*. Upon contact with macrophages, however, *Eh*CP-A1 polarized to the macrophage contact point (**Fig 7**). *Eh*CP-A4 displays a similar diffuse pattern in the absence of macrophages, and similarly polarizes to the macrophage junction upon contact. Quantification of the polarization of *Eh*CP-A1 and *Eh*CP-A4 is included on the right-hand side (**Fig 7**). Positive values indicate asymmetrical staining in the axis towards the macrophages, whereas null values are indicative of a symmetrical distribution of the staining patterns throughout *Eh*. Therefore, *Eh*CP-A1 and *Eh*CP-A4 staining shows polarization towards the macrophage contact interface.

### *Eh*-triggered caspase-6 activation triggers IL-1β secretion by macrophages

**Fig 8:**
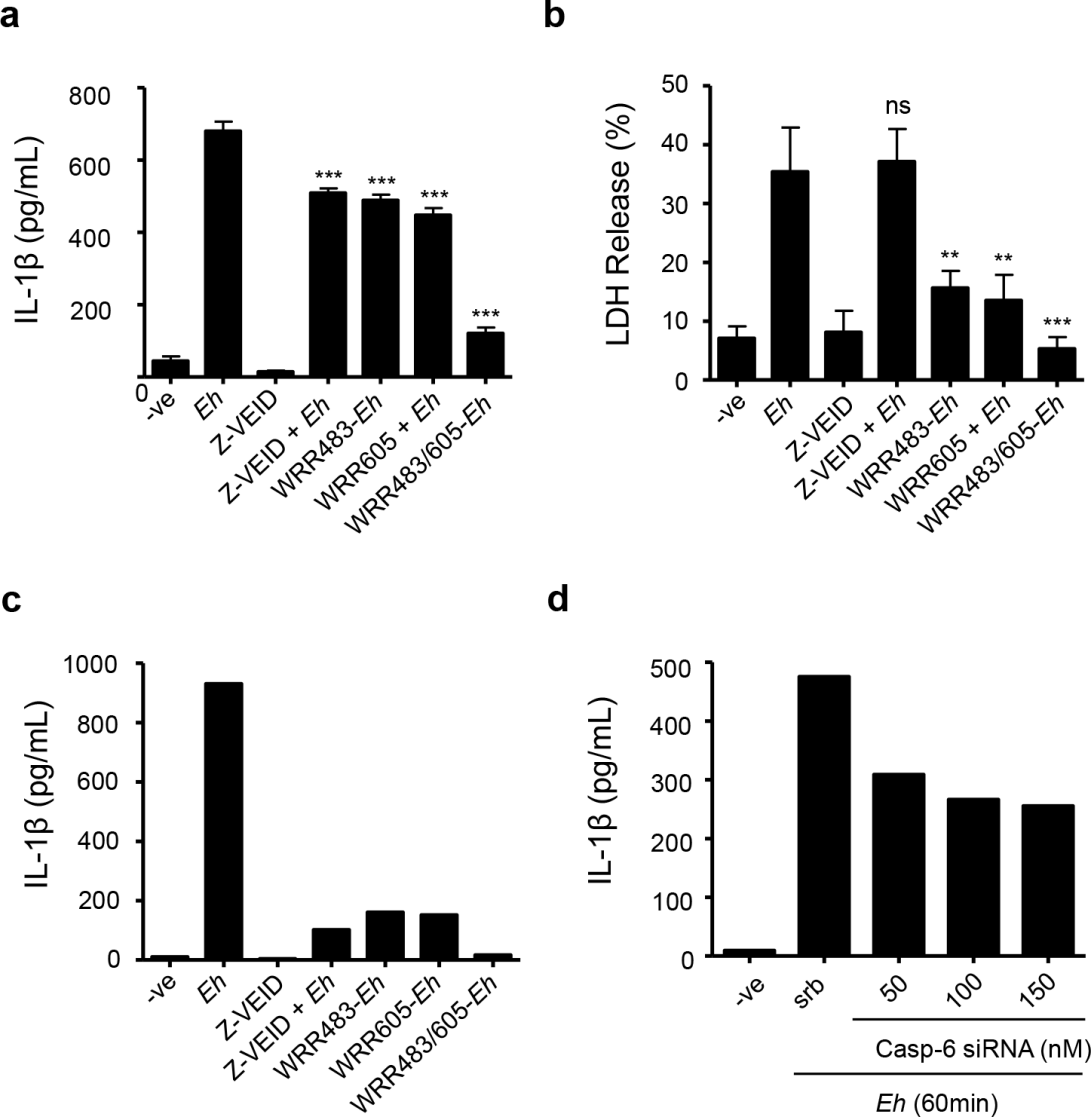
Inhibition of caspase-6 or *Eh*CP-A1 and *Eh*CP-A4 reduces IL-1β secretion by macrophages in response to *Eh*. Caspase-6 was inhibited by pre-treatment of macrophages with 50mM of Z-VEID, or DMSO as control, prior to incubation with *Eh* (10:1 ratio) for 60 min. *Eh*CP-A1 and *Eh*CP-A4 were inhibited by pre-treatment of *Eh* with 20 μM of WRR483, or WRR605, or both, for 30 minutes prior to incubation with macrophages for 60 min. Both IL-1β secretion (**a**) and LDH release (**b**) were measured in media by ELISA. Results are representative of three independent experiments. Statistical significance was assessed by Student’s *t*-test (**p<0.003, ***p<0.0002). (**c**) Samples were also analyzed by Luminex addressable laser bead-based immunoassay (Human Focused 13-Plex Discovery Assay). (**d**) Caspase-6 involvement in IL-1β secretion by THP-1 cells was also assessed by knockdown with the indicated concentrations of caspase-6 siRNA. Scramble siRNA was used as a negative control. IL-1β levels shown are representative of two independent experiments (**c, d**).

As caspase-6-mediated cleavage of cytoskeletal proteins of macrophages is an early event upon contact with *Eh*, we next determined whether it impacted pro-inflammatory cytokine secretion. We hypothesized that caspase-6-mediated cleavage of cytoskeletal proteins may participate to the macrophage inflammatory response upon contact with *Eh*. To determine this, we first measured IL-1β pro-inflammatory cytokine secretion in macrophages pre-treated with the caspase-6 inhibitor, or DMSO control, for 60 min followed by incubation with *Eh* for 60 min. Pre-treatment of macrophages with Z-VEID-fmk significantly (*p*<0.0001) inhibited IL-1β release upon contact with *Eh* (**Fig 8A**). To evaluate the contribution of cell death in active secretion of IL-1β as opposed to passive release following cell death, we also measured LDH levels in media as an indicator of cell death. LDH levels corresponded to up to (35%) of LDH levels from the positive lysis control, indicating that a portion of macrophages was killed by *Eh* following incubation though the majority was still intact (**Fig 8B**). Z-VEID pre-treatment of macrophages prior to *Eh* incubation did not significantly alter LDH release (**Fig 8B**). Therefore, caspase-6 activation does not seem to significantly contribute to cell death within the first 60 min. To quantify the contribution and upstream involvement of *Eh*CP-A1 and *Eh*CP-A4 to IL-1β secretion, we incubated macrophages with *Eh* pre-treated with WRR483, WRR605, or both. IL-1β secretion by macrophages was significantly decreased when stimulated with either inhibitor (*p*<0.0001), and more so when used in combination (**Fig 8A**). LDH release was significantly inhibited by WRR483 and WRR605 (*p*<0.003), or both (*p*<0.0002) indicating these CPs may contribute to macrophage cell death, although this may be mediated through a mechanism independent of caspase-6 (**Fig 8B**). With the use of a highly sensitive Luminex human focused 13-plex-discovery assay we determined that no other cytokine other than IL-1β was secreted at detectable levels, or dependent on caspase-6 (**Fig 8C**). IL-2, IL-4, IL-6, IL-12 and IL-13 secretion was undetectable by Luminex upon stimulation of THP-1 by *Eh* trophozoites. IL-5, GM-CSF, IFN-γ, MCP-1 and TNF-α were secreted at very low levels (less than 20pg/ml). We further confirmed a role for caspase-6 in THP-1-mediated secretion of IL-1β upon stimulation by *Eh*. Consistent with results obtained with the Z-VEID inhibitor, we found that silencing of caspase-6 by siRNA decreased IL-1β secretion in a concentration-dependent manner (**Fig 8D**). Taken together, these data show that *Eh*CP-A1, *Eh*CP-A4, and caspase-6 participate in the pro-inflammatory response of macrophages to *Eh*.

## Discussion

In this study, we determined the molecular events that take place at the intercellular junction between macrophages and *Eh*. We observed cytoskeletal rearrangement consistent with *Eh* engagement of cell-surface receptors, where microtubules reorient towards the target *Eh*, as well as actin accumulation at the contact point. This is reminiscent of many immune cell interactions such as the immune synapse and the phagocytic cup where receptor engagement, including integrins, induces microtubule polarization towards the target as well as actin accumulation at the site of contact [48, 49]. This polarization could be a means to deliver key signaling complexes at the contact site, including the NLRP3 inflammasome which has been shown to co-localize with actin at the contact site [29]. The α_5_β_1_ integrin as well as phosphorylated paxillin was also shown to localize at the contact site indicating downstream signaling occurred upon interaction [29]. We thus sought to further investigate post-translational regulation of key cytoskeletal-associated proteins upon macrophage contact with *Eh*. While we hypothesized that these proteins would be subjected to phosphorylation, what we observed was that these proteins were rapidly subjected to cleavage. Cleavage of talin, Pyk2 and paxillin by calpains or caspase-3 has been reported in the context of adhesion disassembly and apoptosis [40, 41, 50–52]. We cannot, however, preclude the fact that phosphorylation may precede cleavage and actually make these proteins susceptible to cleavage by inducing conformational change [38, 53]. This is supported by previous studies showing that serine phosphorylation of paxillin promotes its cleavage during cell migration [54]. Interference with the host cell cytoskeleton may be a mechanism by which *Eh* ingest host cells, either by trogocytosis or by phagocytosis. Trogocytosis is observed rapidly upon contact with live host cells that exhibit cell membrane deformability [31, 32]. Modification of the host cell cytoskeleton upon contact may represent the early molecular mechanisms allowing for cell deformability and subsequent ingestion of membrane portions by *Eh*. The process of trogocytosis, however, has not yet been reported in macrophages.

Our data unveil a new role for caspase-6 in the alteration of cytoskeletal-associated proteins, as well as in the macrophage inflammatory response to *Eh*. We show that cleavage of talin, Pyk2 and paxillin upon contact with *Eh* was mediated by caspase-6 activation as inhibition of caspase-6 by Z-VEID prevented their cleavage. In the latter, caspase-6 was shown to concentrate proximally to points of contact with *Eh*. Although caspase-6 is classified as an effector caspase along with caspase-3 and caspase-7, there is increasing evidence that its role extends beyond apoptosis [55]. A role for caspase-6 in B-cell activation and differentiation, and more recently in macrophage activation, has been shown [56, 57]. Moreover, caspase-6 is ubiquitously expressed and the highest levels of pro-caspase-6 are observed in fetal and adult colon [58]. Therefore, further studies in the role of caspase-6 in gut inflammatory responses are warranted to assess whether this mechanism is unique to amebiasis or whether caspase-6 plays a broader role in triggering cytokine secretion and cytoskeletal remodeling.

The caspase-6-mediated cleavage of cytoskeletal-associated proteins was only observed when macrophages were incubated with live *Eh*. Similar results were observed with NLRP3 inflammasome activation upon ligation of *Eh*CP-A5 and Gal-lectin. Soluble *Eh*CP-A5 or Gal-lectin alone was not sufficient to induce NLRP3 and caspase-1 activation and subsequent IL-1β release [29]. This likely reflects the importance that *Eh* factors to be delivered to the intercellular junction in a directed manner allowing for saturation of macrophage receptors to trigger downstream signaling cascades. We propose that this mechanism allows macrophages to distinguish between interactions of live invading *Eh* in comparison to sampling of soluble components of the gut lumen. The escalating pro-inflammatory response ensuing detection of live pathogens thus reflects the tailoring of the reaction against tissue-invading pathogens. The requirement for direct cell contact may also reflect the necessity of *Eh* to attach to host cells to release intracellular contents at the intercellular junction. In fact, *Eh*CP-A1 and *Eh*CP-A4, which we show are necessary for triggering cleavage of cytoskeletal-associated proteins, localize to intracellular vesicles in *Eh* [20, 44]. *Eh*CP-A1 localizes to large cytoplasmic vesicles whereas *Eh*CP-A4 is contained in acidic vesicles in *Eh*, yet has been shown to be released in culture medium as well as in the microenvironment *in vivo*. *Eh*CP-A4 undergoes autocatalytic activation at acidic pH, provided by acidic vesicles, yet its optimal proteolytic activity is observed at physiological pH [44]. By staining the surface of non-permeabilized cells, we showed that both *Eh* cysteine proteinases are found at the cell surface, and polarized to the macrophage contact site. This supports a model by which these cysteine proteinases are delivered at the junction site in a defined manner, and have a role in triggering the macrophage inflammatory response. Future studies on the dynamics of *Eh*CP-A1- and *Eh*CP-A4-containing vesicles upon contact with host cells are necessary to uncover the mechanism of release in the microenvironment.

**Fig 9:**
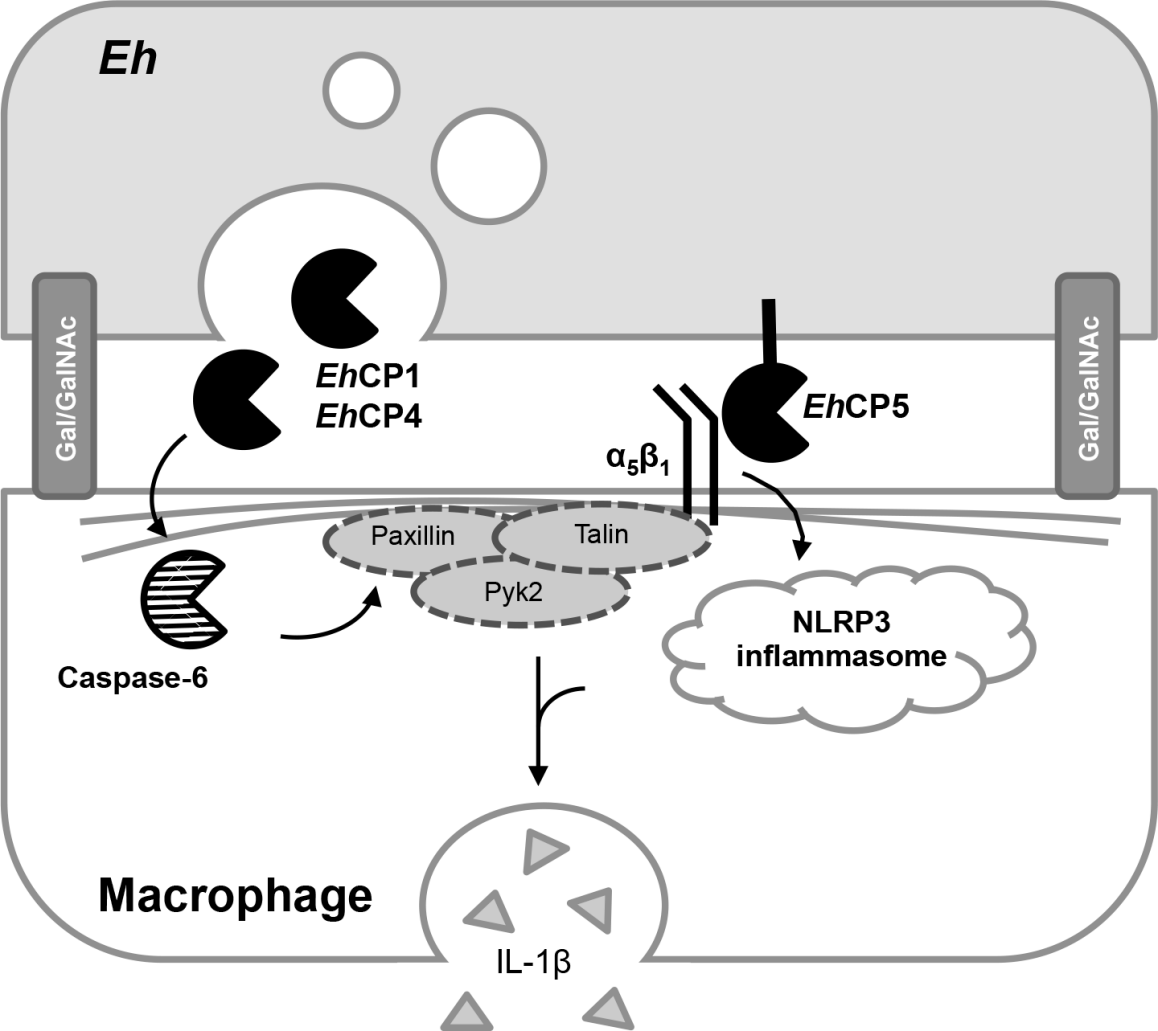
Schematic representation of the *Eh*-macrophage interacting proteins at the intercellular junction. Contact between *Eh* and macrophages are initiated by high affinity binding with the Gal-lectin. *Eh*CP-A5, which is expressed at the surface of *Eh*, has been shown to bind to the α_5_β_1_ integrin at the surface of macrophages through the RGD motif. This in turn activates the NLRP3 inflammasome and triggers IL-1β secretion by macrophages. In parallel, *Eh*CP-A1 and *Eh*CP-A4, which are localized in intracellular vesicles, are released at the intercellular junction. Through an unidentified mechanism, these cysteine proteinases trigger caspase-6 activation and subsequent cleavage of cytoskeletal-associated proteins paxillin, Pyk2 and talin. This arm of the *Eh*-macrophage interaction contributes to enhance IL-1β release.

Our data unveil new roles for both *Eh*CP-A1 and *Eh*CP-A4 in eliciting pro-inflammatory responses by macrophages. Interestingly, *Eh*CP-A4 expression is significantly higher in HM-1:IMSS than in the less virulent Rahman strain, whereas *Eh*CP-A1 and *Eh*CP-A5 transcription levels are similar between those two strains [45]. Similarly, *Eh*CP-A1 release in medium is significant only for HM-1:IMSS compared to *E*. *dispar* and the less virulent L6 *Eh* clone [23]. *Eh*CP-A1 expression has been shown to increase almost twofold following invasion in a mouse model of amebic colitis, whereas *Eh*CP-A5 expression was unchanged [46]. Contrary to our initial hypothesis that *Eh*CP-A5 would have a central role in regulating key cytoskeletal-associated proteins as it binds to macrophage integrin, it was not necessary for triggering cleavage of talin, Pyk2 or paxillin. Rather, the finding of another mechanism that relies on direct contact between macrophage and *Eh*, and that culminates in IL-1β release further reinforces that the intercellular junction is a hot spot for molecular events that shape the subsequent pro-inflammatory response during amebiasis. We propose an integrated model (**Fig 9**) by which contact between live *Eh* and macrophages is first established by binding via the Gal-lectin at the *Eh* cell surface as well as *Eh*CP-A5 binding to macrophage α_5_β_1_ integrin. We hypothesize that contact mediates the release of *Eh*CP-A1 and *Eh*CP-A4 from *Eh* in a polarized fashion at the intercellular junction. The *Eh*CP-A5/integrin axis mediates NLRP3 inflammasome activation whereas *Eh*CP-A1 and *Eh*CP-A4 mediate caspase-6 activation and subsequent cytoskeletal-associated protein cleavage. Together, these pathways culminate in potent IL-1β release from macrophages. The molecular mechanisms linking some of these events remain to be addressed. For example, the requirements for *Eh*CP-A1 and *Eh*CP-A4 release and interaction with macrophages as well as the signaling events that culminate in caspase-6 activation remain unknown. The consequence of post-translational modification of cytoskeletal-associated proteins in remodeling of the macrophage cytoskeleton upon contact with *Eh* also needs to be examined. Nonetheless, this study underlines the fact that the intercellular junction is a hotspot for initiation of the inflammatory response to *Eh* and further investigation will unravel mechanistic pathways that will provide a better understanding of macrophage and amebic biology.

## Methods

### Reagents

E-64 was obtained from Sigma-Aldrich. Caspase inhibitors were obtained from Enzo Life Sciences. Anti-paxillin (clone 349) was purchased from BD Biosciences. Anti-Pyk2 and anti-GAPDH were acquired from EMD Millipore. Anti-talin was obtained from Santa Cruz Biotechnologies. Anti-caspase-6 and anti-lamin were acquired from Cell Signaling Technology. Anti-mouse and anti-rabbit conjugated to HRP were from Jackson ImmunoResearch. Cell Tracker Blue, Cell Tracker Orange, A647-conjugated phalloidin and A488-conjugated anti-tubulin were obtained from Life Technologies. Recombinant human caspase-6 and the Z-VVR-AMC substrate were purchased from Enzo Life Sciences. The Z-Arg-Arg-pNA.2 HCl (Z-RR) substrate was purchased from Bachem. The FAM-FLICA caspase-6 assay kit was purchased from ImmunoChemistry Technologies. Transfection was carried out with the INTERFERin (Polypus) and caspase-6 siRNA (Santa Cruz Biotechnologies) or scramble siRNA (GE Dharmacon). Antibodies to *Eh*CP1 and *Eh*CP4, and WRR483 and WRR605 inhibitors were a gift from Dr. Sharon Reed, University of California, San Diego). Human IL-1β secretion in cell culture was quantified by ELISA (R&D Systems). LDH levels were measured using the CytoTox-ONE homogeneous membrane integrity assay (Promega).

### *E*. *histolytica* culture

*Eh* trophozoites of the highly virulent HM-1:IMSS strain were grown axenically in TYI-S-33 medium supplemented with 100U/ml penicillin and 100μg/ml streptomycin sulfate as described previously [59]. *Eh*CP-A5-deficient trophozoites were a gift from Dr. David Mirelman (Weizmann Institute of Science, Rehovot, Israel) and identically cultured. Virulence was maintained by regular sub passage in gerbil livers as previously described [60]. Trophozoites were harvested during log-phase growth by centrifugation at 200 x g at 4°C for 5 minutes and resuspended in RPMI.

### Cell preparation and stimulation

The THP-1 monocytic cell line (ATCC, Manassas, VA) was cultured in RPMI with 10% FBS, 10mM HEPES, 50μM 2-mercaptoethanol, 100U/ml penicillin and 100μg/ml streptomycin sulfate in a humidified incubator with 5% CO_2_. THP-1 cells were differentiated into macrophages by seeding 8 x 10^5^ cells per well in 12-well plates in complete medium supplemented with 50ng/ml phorbol-12-myristate-13-acetate (PMA) overnight. Bone marrow derived macrophages (BMM) were cultured from bone marrow cells of C57BL/6 mice and cultured for 6 days in complete medium supplemented with 30% L929-cell supernatant. Cells were then plated at 5 x 10^5^ cells per well in 12-well plates in complete medium. On the day of experiment, BMDM were treated with 1μg/ml LPS for 3.5h prior to stimulation with *Eh*. The T84 epithelial cell line (ATCC, Manassas, VA) was cultured in DMEM/F-12 with 10% FBS, 10mM HEPES, 100U/ml penicillin and 100μg/ml streptomycin sulfate. For experiments, cells were seeded at 3 x 10^5^ cells per well in 12-well plates and grown to a confluent monolayer.

For stimulation with *Eh*, cells were incubated at 37°C with in serum-free RPMI for the indicated times. *Eh* were washed off with cold PBS and cells were lysed in lysis buffer (1% Triton X-100, 20mM Tris, 100mM NaCl, 1mM EDTA, 200mM orthovanadate, sodium fluoride, 0.1% SDS, PMSF, leupeptin, aprotinin, and protease inhibitor cocktail). For inhibitor treatment of macrophages, PMA-differentiated THP-1 cells were pre-treated with inhibitors for 1h at 37°C in serum-free RPMI prior to incubation with *Eh*. For inhibition of adhesion, *Eh* were incubated with 55mM D-galactose, or glucose as control, for 5 min prior to incubation with THP-1 macrophages. For irreversible inhibition of total cysteine proteinase activity of *Eh*, *Eh* were incubated overnight in medium with 100μM of E-64, as previously described [61]. For inhibition of specific *Eh*CP activity, *Eh* were incubated with 20μM of the *Eh*CP-A1 inhibitor (WRR483), 20μM of the *Eh*CP-A4 inhibitor (WRR605), or both, for 30 min and then washed prior to incubation with THP-1 macrophages.

### *In vitro* enzymatic assays

To evaluate enzymatic specificity of the WRR483 and WRR605 inhibitors, known *Eh*CP-A1 and *Eh*CP-A4 substrates (Z-Val-Val-Arg and Z-Arg-Arg, respectively) were incubated for 0 to 20 minutes at 37°C with *Eh* trophozoites pre-treated with either WRR483, WRR605, both, or E-64 as described above. Cleavage of the Z-VVR-AMC fluorogenic substrate was detected at the 460nm wavelength, and cleavage of the chromogenic Z-RR-pNA.2 HCl substrate was detected at 405nm. For *in vitro* cleavage of proteins by caspase-6, THP-1 cells were lysed with lysis buffer as described above, and 10μg of lysate was incubated with anti-paxillin, anti-lamin or anti-mouse IgG for 15 min and 1.5 h with Protein A/G plus agarose. The immunoprecipitate was then incubated with 2 units of recombinant caspase-6 for 16 h at 37°C. Immunoprecipitates were then resuspended in reducing sample buffer and boiled prior to loading onto SDS-PAGE gel.

### Caspase-6 siRNA

THP-1 cells were differentiated overnight with PMA prior to transfection as described above. Cells were transfected with caspase-6 siRNA, or scramble siRNA as a control, with the INTERFERin reagent, as per the manufacturer’s protocol. A total of 8uL of INTERFERin reagent was used for each transfection with caspase-6 siRNA, at the indicated concentrations, or scrambled siRNA as the control. Media was replaced with complete RPMI 24 h later. *Eh* stimulation was performed 48 h following siRNA transfection for 2 min for analysis by Western blot, or 60 min for assessment of IL-1β secretion.

### Western blots

Equal amounts of lysate were loaded on SDS-PAGE gel followed by transfer onto polyvinylidene difluoride (PVDF) membrane and blocking in 5% skim milk. Western blots were done using the indicated primary antibody and appropriate HRP-conjugated secondary antibody, and visualized with either SuperSignal Chemiluminescence Reagents (Pierce Biotechnology) or ChemiLucent ECL detection (EMD Millipore). When sequential Western blots were performed, PVDF membranes were incubated with stripping buffer (25mM Glycine, 1% SDS, pH 2.0) for 30 min at room temperature, followed by extensive washing and blocking with 5% skim milk. None of the antibodies used cross-reacted with proteins in *Eh* lysate, as verified by Western blot.

### Confocal microscopy

For visualization of *Eh* contact with THP-1, THP-1 cells were PMA-differentiated and seeded on glass coverslips overnight. *Eh* were stained with Cell Tracker Blue according to the manufacturer’s protocol prior to incubation with THP-1 cells for 15 min at 37°C. After incubation, cells were gently washed with PBS, fixed with 4% paraformaldehyde for 10 min, permeabilized with 0.1% NP-40 in PBS followed by staining with Alexa-488-conjugated anti-tubulin, or isotype control, and with Alexa-647-conjugated phalloidin. For visualization of caspase-6 activation in THP-1 cells following stimulation with *Eh*, *Eh* were stained with Cell Tracker Orange according to the manufacturer’s protocol prior to incubation with PMA-differentiated THP-1 cells for 15 min at 37°C. Slides were fixed and permeabilized as described above. Slides were stained with the FAM-FLICA caspase-6 probe as per the manufacturer’s protocol for 60 min, and with DAPI for 15 min for visualization of nuclei. Coverslips were then washed with the provided apoptosis washing buffer and mounted onto microscope slides with ProLong antifade (Thermofisher) and visualized using the Olympus IX81 FV1000 Fluoview Laser Scanning Confocal.

For staining of *Eh*CP-A1 and *Eh*CP-A4, THP-1 were seeded onto glass coverslips and incubated with *Eh* as described above. Slides were fixed in 4% paraformaldehyde for 15 min but not permeabilized to detect only extracellular surface-bound *Eh*CPs. Following extensive washing post-fixation with 0.2% Tween 20 in PBS, slides were blocked with 5% normal donkey serum and subsequently incubated overnight with primary antibodies against *Eh*CP-A1 and *Eh*CP-A4 at 4°C. Slides were then washed and incubated for 60 min with the appropriate secondary antibodies (Donkey anti-Rabbit 1:250 and donkey anti-Chicken IgY 1:250) and DAPI at room temperature. Quantification of the abundance of activated caspase-6 at the THP-1/*Eh* interface was done in ImageJ as previously published^30^. Quantification of *Eh*CP polarization was performed in ImageJ by measuring the integrated density of the front half of *Eh* and rear facing half, expressing this as a ratio adjusted for total area size. Figures for confocal images were created in Adobe Photoshop CS5.

### Flow cytometry

PMA-differentiated THP-1 cells were harvested with TrypLE (ThermoFisher Scientific) for 5 min at 37°C followed by washing with serum-containing media. 10^6^ THP-1 cells were incubated with *Eh* (10:1) and stained for 60 min with the FAM-FLICA caspase-6 reagent as per the manufacturer’s protocol. One sample of cells was also treated with STS for 3 h as a positive control for caspase-6 activation. A sample with *Eh* cells only was used to establish exclusion gates based on forward and side scatter and FAM-FLICA caspase-6-stained *Eh* showed no non-specific staining of trophozoites. Samples were washed with the washing buffer provided in the FAM-FLICA caspase-6 assay kit and fixed with 4% paraformaldehyde prior to acquisition with the BD FACSCanto Flow Cytometer. Data analysis was done with FlowJo.

### Cytokine and LDH detection

PMA-differentiated THP-1 cells were incubated with *Eh* (10:1) in serum-free media for 60 min at 37°C. Supernatants were collected, centrifuged at 3000 x g for 5 min to spin out cells, and transferred to new eppendorf tubes. Quantification of IL-1β and LDH levels for each sample were measured by ELISA as per the manufacturer’s protocol. Samples were also analyzed by Luminex addressable laser bead-based immunoassay (Human Focused 13-Plex Discovery Assay; Eve Technologies, Calgary, AB).

### Animals

C57BL/6 mice were obtained from Charles River. *Asc*^*-/-*^ mice, bred onto a C57BL/6 background, were obtained from Drs. Muruve and Beck (University of Calgary). Femurs and tibia from these mice were used for the growth of bone-marrow-derived macrophages (BMM).

### Ethics statement

The Health Sciences Animal Care Committee from the University of Calgary, have examined the animal care and treatment protocol (AC17-0017) and approved the experimental procedures proposed and certifies with the applicant that the care and treatment of animals used was in accordance with the principles outlined in the most recent policies on the “Guide to the Care and Use of Experimental Animals” by The Canadian Council on Animal Care.

### Statistics

Experiments are representative of at least three independent experiments unless otherwise noted. Statistical significance between groups was assessed by Student’s *t*-test whereby p<0.01 was considered significant. For quantification of confocal images, a minimum of 6 images was used for each condition.
